# Supplementary figures and images for: Cryopreservation of human mucosal tissues
Source: PLoS One. 2018 Jul 30;13(7):e0200653. doi: 10.1371/journal.pone.0200653 (PMC6066204; doi:10.1371/journal.pone.0200653)

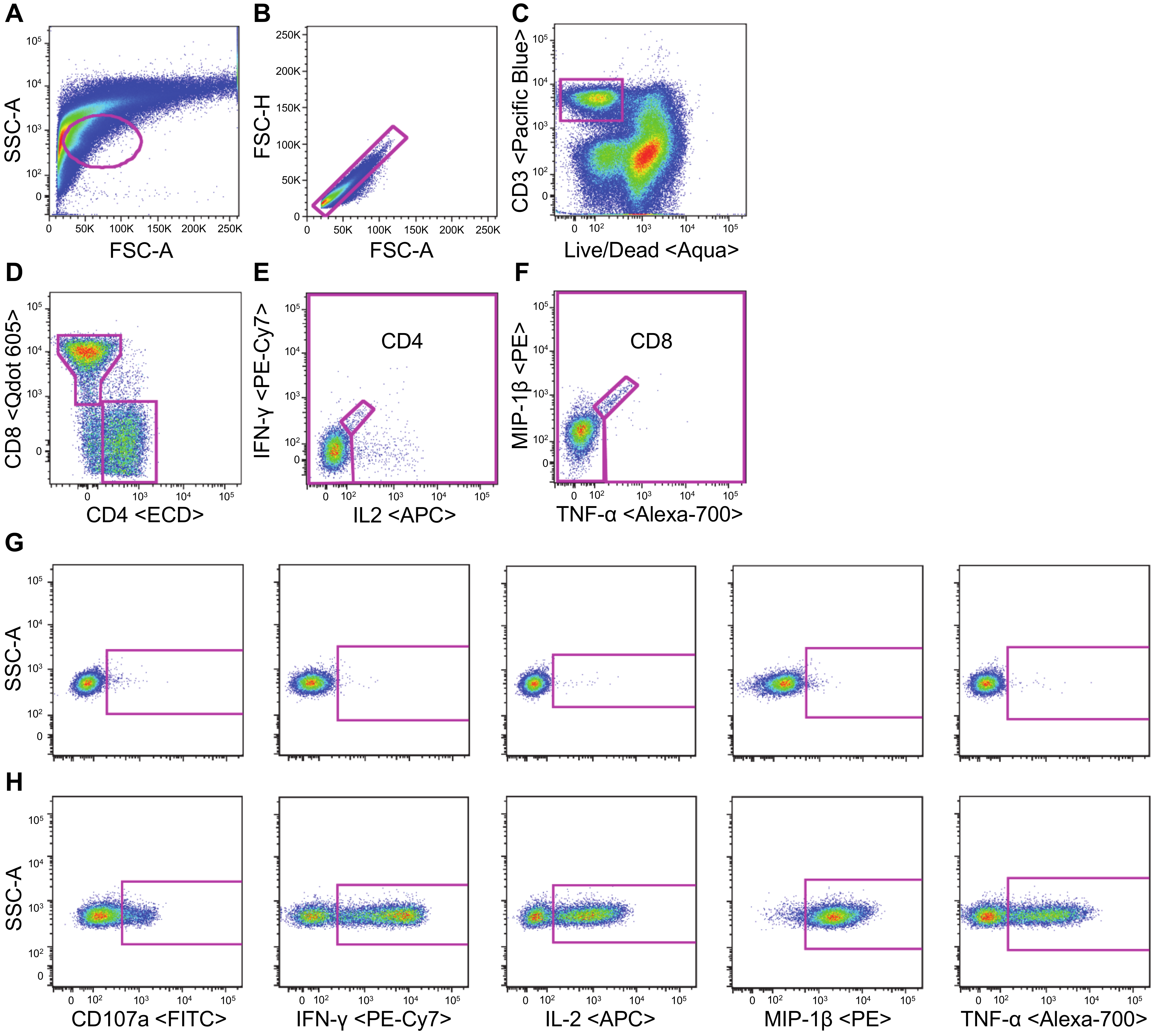

Supplement: S2 Fig — A, Selection of lymphocyte-like cells by forward and side scatter. B, Exclusion of doublets. C, Identification of live CD3+ T cells. D, Separation of T cells into CD4 and CD8 subsets. E, Exclusion of non-specific staining from CD4 cells. All events are included in this gate except for those inside the diagonal box. F, Exclusion of non-specific staining from CD8 cells. G, Identification of cytokine- or CD107a-expressing cells in the unstimulated condition. H, Identification of cytokine- or CD107a-expressing cells in the PMA/ionomycin-stimulated condition. “FSC” and “SSC” refer to forward and side scatter, with “-A” indicating area and “-H” indicating height. Cytokines measured were interferon-γ (IFN- γ), interleukin-2 (IL-2), macrophage inflammatory protein (MIP)-1β, and tumor necrosis factor-α (TNF-α). “APC” indicates allophycocyanin. (TIFF) [file pone.0200653.s002.tiff]
