# Supplementary material for: Cryopreservation of human mucosal tissues
Source: PLoS One. 2018 Jul 30;13(7):e0200653. doi: 10.1371/journal.pone.0200653 (PMC6066204; doi:10.1371/journal.pone.0200653)
Supplement: S1 Table — Reagent information for all antibodies. (DOCX) [file pone.0200653.s003.docx]

| *Antibody* | *Fluorochrome* | *Clone* | *Company* | *Catalog #* | *Panel* |
| --- | --- | --- | --- | --- | --- |
| CD45 | APC-Cy7 | 2D1 | BD | 557833 | Colorectal phenotyping |
| CD3 | BV605 | SK7 | BD | 563219 | Colorectal phenotyping |
| CD4 | PE-Cy7 | SK3 | BD | 557852 | Colorectal phenotyping |
| CD8 | PE | SK1 | BD | 340046 | Colorectal phenotyping |
| CD33 | BV711 | WM53 | BD | 563171 | Colorectal phenotyping |
| CD66b | BV421 | RA3-6B2 | BD | 562940 | Colorectal phenotyping |
| CD13 | PE-CF594 | WM15 | BD | 562491 | Colorectal phenotyping |
| CD3 | Pacific Blue | UCHT1 | BD | 558117 | Colorectal ICS |
| CD4 | ECD | SFCI12T4D11 | Beckman | 6604727 | Colorectal ICS |
| CD8 | QDot 605 | 3B5 | Life Technologies | Q10009 | Colorectal ICS |
| CD107a | FITC | H4A3 | BD | 555800 | Colorectal ICS |
| Interferon-γ | PE-Cy7 | B27 | BD | 557643 | Colorectal ICS |
| Tumor necrosis factor-α | Alexa 700 | MAb11 | BD | 557996 | Colorectal ICS |
| Interleukin 2 | APC | 25723.11 | BD | 341117 | Colorectal ICS |
| Macrophage inflammatory protein-1β | PE | D21-1351 | BD | 550078 | Colorectal ICS |
| CD28 | Pure | L293 | BD | 348040 | Colorectal ICS |
| CD49d | Pure | L25 | BD | 340976 | Colorectal ICS |
