# Supplementary material for: Cryopreservation of human mucosal tissues
Source: PLoS One. 2018 Jul 30;13(7):e0200653. doi: 10.1371/journal.pone.0200653 (PMC6066204; doi:10.1371/journal.pone.0200653)
Supplement: S1 Text — (DOCX) [file pone.0200653.s004.docx]

Cryopreservation of mucosal biopsies

# Freezing procedure

Reagents needed:

- 10% DMSO in FBS (1 mL)
  - 0.9 mL heat inactivated fetal bovine serum
  - 0.1 mL DMSO
- Cryovials

*Procedure*

1. Prepare the 10% DMSO in FBS and chill at 4^o^C for 30 minutes.
2. Place 200 µL of the DMSO/FBS solution in each cryovial
   1. Note: Larger volumes may be used with multiple biopsies. E.g. use 1 mL with 5-10 biopsies
3. Place a biopsy in the cryovial. If necessary, add more 10% DMSO in FBS to cover.
4. Close cryovial and place in a Mr. Frosty to freeze at -80^o^C overnight.
5. For storage, place samples in a liquid nitrogen freezer until needed.

# Thawing procedure

Reagents needed:

- Culture medium at room temperature (about 25^o^C)

*Procedure*

1. Remove the cryovials from the liquid nitrogen freezer, but keep them on liquid nitrogen in a pan or other device for carrying liquid nitrogen until ready to thaw.
2. Transfer cryovials to a 37^o^C water bath and agitate until thawed.
3. Transfer biopsies with forceps into a large volume (5 mL) of room temperature culture medium.
4. After 10 minutes, the biopsies are ready for use.

Vitrification of mucosal biopsies

# Freezing procedure

Reagents needed:

- 1X Vitrification medium (5 mL)
  - 1 mL EG (ethylene glycol)
  - 1 mL DMSO
  - 600 µL fetal bovine serum
  - 2.4 mL phosphate buffered saline
- 0.5X Vitrification medium (5 mL)
  - 0.5 mL EG
  - 0.5 mL DMSO
  - 800 µL fetal bovine serum
  - 3.2 mL phosphate buffered saline
- Aluminum foil pieces (one per biopsy)
  - Cut rectangular pieces of foil that are just narrower than the width of a cryovial and just shorter than the height of the cryovial up to the threads of the cap
  - Check to see that each foil pieces goes easily into a cryovial without catching.
- Six-well plate

*Procedure*

1. Prepare explants/biopsies 5mmx5mm or smaller.
2. Place 5 mL of the 0.5X vitrification medium in a well of a six-well plate. Place 5 mL of the 1X vitrification medium in a second well of a six-well plate.
3. Refrigerate the six-well plate for 30 min.
4. Prepare a pan of liquid nitrogen with absorbent cloth and place a cryovial rack so the bottom of the cryovials are immersed in liquid nitrogen. Make sure that there is a part of the pan where enough liquid nitrogen is exposed that you can immerse the biopsies at least one inch into the liquid.
5. Place empty cryovials in a rack in the liquid nitrogen container so they are cool when you put the biopsy in.
6. Remove the six-well plate from the refrigerator and place in a biosafety cabinet.
7. Transfer biopsies with forceps into the 0.5X solution.
8. After 5 minutes, transfer biopsies with forceps into the 1X solution.
9. After 5 minutes, briefly blot biopsies individually on a kimwipe or sterile wipe. This is to remove the vitrification medium that is coating the biopsies.
10. Place a biopsy close to the edge at the narrow end of a pre-cut piece of aluminum foil.
11. Pick up the other end of the aluminum foil with forceps.
12. Plunge the entire foil (and some of the forceps) into liquid nitrogen.
13. After about 10 seconds, when the bubbling has subsided, place the foil with the biopsy frozen to it into a cryovial (precooled in liquid nitrogen).
    1. Note: 3-4 biopsies can typically fit into one cryovial. Foil could be cut differently to allow more to fit.
14. Cap the cryovials and store in a liquid nitrogen freezer until needed.

# Thawing procedure

Reagents needed:

- 0.5X thawing medium (5 mL)
  - 0.5 mL EG
  - 0.5 mL DMSO
  - 4 mL R10 or other culture medium
- Six-well plate

*Procedure*

1. Place 5 mL of the thawing medium in a well of a six-well plate. Place 5 mL of plain culture medium into a second well and keep the plate at room temperature.
2. Remove the cryovials from the liquid nitrogen freezer, but keep them on liquid nitrogen in a pan or other device for carrying liquid nitrogen.
3. While keeping the cryovials on liquid nitrogen, unscrew the caps.
4. Use forceps to remove one piece of aluminum foil/biopsy from a cryovial.
5. Quickly move the foil out of the liquid nitrogen pan and place it, biopsy side down, in the well with the thawing medium and shake until biopsy detaches (~5 seconds)
   1. Several samples from the same donor can be thawed and transferred at the same time (a minute more or less of time in the thawing media and culture medium doesn’t make a difference)
6. After 10 minutes, use forceps to transfer the biopsy to the culture medium.
7. After 10 minutes in the culture medium, the biopsy is ready for use.
