# Supplementary material for: Cryopreservation of human mucosal tissues
Source: PLoS One. 2018 Jul 30;13(7):e0200653. doi: 10.1371/journal.pone.0200653 (PMC6066204; doi:10.1371/journal.pone.0200653)
Supplement: S1 File — (PDF) [file pone.0200653.s005.pdf]

Cryopreservation of human mucosal tissue: statistics

Contents

Summary of all tissue viability assessments . . . 2
HIV-1 infection of vaginal tissues . . . 3
Cell yields from cryopreserved and vitrified colorectal biopsies . . . 4
HIV-1 infection of colorectal tissues . . . 5
HIV-1 infection of ectocervical tissues . . . 5
Comparison of cryopreserving whole biopsies and isolated cell suspensions . . . 6
Microbicide drug concentrations in cryopreserved tissue . . . 12

Summary of all tissue viability assessments

Table A: Percent of fresh alamar blue fluorescence

Condition	Mean	95% CI	n
Cryopreserved	103.51	[87.81, 119.2]	10
Vitrified	99.76	[84.07, 115.44]	10
Formalin	8.25	[4.27, 12.24]	10

Table B: Non-inferiority testing of alamar blue fluorescence. Test for difference by paired, two-sided t-test with null hypothesis of no difference. Test for non-inferiority by paired, one-sided t-test, with null hypothesis of difference of 20%.

Comparison	Difference p	Non-inferiority p
Fresh vs. cryopreserved	0.97	6.56e-03
Fresh vs. vitrified	0.56	0.02

HIV-1 infection of vaginal tissues

Table C: Hazard ratios relative to fresh tissue from Cox proportional hazards model. Overall likelihood ratio test p = 0.96

Condition	Hazard Ratio	95% CI	p
Cryopreservation	1.03	[0.56, 1.92]	0.92
Vitrification	0.95	[0.51, 1.75]	0.87

Cell yields from cryopreserved and vitrified colorectal biopsies

Table D: Live colorectal cell yield (millions)

Condition	Mean	95% CI	n
Cryopreserved	6.82	[4.44, 9.21]	5
Vitrified	0.27	[-0.02, 0.56]	5

Table E: Difference in yield of viable cells from cryopreserved samples (millions), paired t-test

CellType	Condition	Mean	95% CI	p	n
Colorectal	Vitrified	-6.55	[-9, -4.1]	1.76e-03	5

Table F: Population characteristics

CellType	Condition	Mean	95% CI	n
Leukocytes of all cells	Cryopreserved	44.50	[32.96, 56.04]	5
Leukocytes of all cells	Vitrified	24.73	[8.04, 41.42]	5
T cells of CD45+	Cryopreserved	54.44	[45.4, 63.48]	5
T cells of CD45+	Vitrified	55.20	[35.44, 74.96]	5
Monocytes of CD3-	Cryopreserved	22.82	[16.57, 29.07]	5
Monocytes of CD3-	Vitrified	37.96	[14.77, 61.15]	5
CD33 myeloid of CD3-	Cryopreserved	2.88	[1.62, 4.13]	5
CD33 myeloid of CD3-	Vitrified	0.64	[0.23, 1.06]	5
Granulocytes of CD3-	Cryopreserved	0.92	[0.41, 1.43]	5
Granulocytes of CD3-	Vitrified	1.12	[0.12, 2.12]	5

Table G: Difference in population characteristics from vitrified, paired t-test

CellType	Condition	Mean	95% CI	p	n
Leukocytes of all cells	Cryopreserved	19.77	[-6.4, 45.95]	0.1	5
T cells of CD45+	Cryopreserved	-0.76	[-21.4, 19.88]	0.92	5
Monocytes of CD3-	Cryopreserved	-15.14	[-36.23, 5.95]	0.12	5
CD33 myeloid of CD3-	Cryopreserved	2.23	[0.99, 3.47]	7.51e-03	5
Granulocytes of CD3-	Cryopreserved	-0.20	[-1.17, 0.76]	0.59	5

HIV-1 infection of colorectal tissues

Table H: Hazard ratios relative to fresh tissue from Cox proportional hazards model. Overall likelihood ratio test p = 0.04

Condition	Hazard Ratio	95% CI	p
10% DMSO	0.70	[0.4, 1.23]	0.22
6% DMSO + 5% EG + 50mM trehalose	0.42	[0.2, 0.86]	0.02

HIV-1 infection of ectocervical tissues

Table I: Hazard ratios relative to fresh tissue from Cox proportional hazards model. Overall likelihood ratio test p = 0.91

Condition	Hazard Ratio	95% CI	p
10% DMSO	1.12	[0.64, 1.97]	0.70
6% DMSO + 5% EG + 50mM trehalose	0.99	[0.52, 1.88]	0.98

Comparison of cryopreserving whole biopsies and isolated cell suspensions

Table J: Colorectal cell viability (%)

Condition	Mean	95% CI	n
Fresh	95.11	[93.32, 96.9]	18
Frozen Biopsies	88.72	[86.25, 91.2]	18
Frozen Suspension	81.12	[76.17, 86.07]	17

Table K: Colorectal cell viability (%), paired samples only

Condition	Mean	95% CI	n
Fresh	94.94	[93.07, 96.81]	17
Frozen Biopsies	89.41	[87.28, 91.55]	17
Frozen Suspension	81.12	[76.17, 86.07]	17

Table L: Colorectal cell viability, ANOVA with Tukey post-test

Comparisons	Estimate	Std. Error	Z value	p
Frozen Biopsies - Fresh	-6.39	2.17	-2.94	0.01
Frozen Suspension - Fresh	-13.99	2.20	-6.35	3.84e-07
Frozen Suspension - Frozen Biopsies	-7.60	2.20	-3.45	3.18e-03

Table M: Colorectal cell viability (paired samples only), repeated measures ANOVA with Tukey post-test

Comparisons	Estimate	Std. Error	Z value	p
Frozen Biopsies - Fresh	-5.53	2.16	-2.56	0.03
Frozen Suspension - Fresh	-13.82	2.16	-6.40	3.04e-10
Frozen Suspension - Frozen Biopsies	-8.29	2.16	-3.84	3.31e-04

Table N: Live colorectal cell yield (millions)

Condition	Mean	95% CI	n
Fresh	13.28	[11.38, 15.19]	18
Frozen Biopsies	9.18	[7.56, 10.8]	18
Frozen Suspension	6.45	[4.95, 7.95]	17

Table O: Live colorectal cell yield (millions), paired samples only

Condition	Mean	95% CI	n
Fresh	13.55	[11.6, 15.49]	17
Frozen Biopsies	9.52	[7.97, 11.06]	17
Frozen Suspension	6.45	[4.95, 7.95]	17

Table P: Colorectal cell number (millions), ANOVA with Tukey post-test

Comparisons	Estimate	Std. Error	Z value	p
Frozen Biopsies - Fresh	-4.11	1.12	-3.66	1.72e-03
Frozen Suspension - Fresh	-6.83	1.14	-6.00	2.42e-06
Frozen Suspension - Frozen Biopsies	-2.72	1.14	-2.40	0.05

Table Q: Colorectal cell number (millions, paired samples only), repeated measures ANOVA with Tukey post-test

Comparisons	Estimate	Std. Error	Z value	p
Frozen Biopsies - Fresh	-4.03	0.84	-4.79	4.32e-06
Frozen Suspension - Fresh	-7.09	0.84	-8.44	0.00e+00
Frozen Suspension - Frozen Biopsies	-3.06	0.84	-3.65	7.93e-04

Table R: Recovery of colorectal cells (%)

Condition	Mean	95% CI	n
Frozen Biopsies	71.35	[58.87, 83.83]	18
Frozen Suspension	49.81	[37.52, 62.11]	17

Table S: Recovery of colorectal cells (%), paired samples only

Condition	Mean	95% CI	n
Frozen Biopsies	73.27	[60.7, 85.85]	17
Frozen Suspension	49.81	[37.52, 62.11]	17

Table T: Cells expressing at least one cytokine (%)

Condition	CellType	Tissue	Mean	95% CI	n
CEF	CD4+	Fresh	0.89	[0.22, 1.56]	7
CEF	CD4+	Frozen Biopsies	1.56	[0.56, 2.55]	7
CEF	CD4+	Frozen Suspension	1.48	[-0.16, 3.13]	4
CEF	CD8+	Fresh	1.68	[-0.06, 3.42]	8
CEF	CD8+	Frozen Biopsies	0.95	[-0.05, 1.96]	8
CEF	CD8+	Frozen Suspension	3.54	[0.15, 6.93]	7
Gag	CD4+	Fresh	0.60	[0.15, 1.06]	7
Gag	CD4+	Frozen Biopsies	2.46	[0.82, 4.1]	7
Gag	CD4+	Frozen Suspension	0.89	[0.07, 1.72]	2
Gag	CD8+	Fresh	6.07	[-1.31, 13.46]	9
Gag	CD8+	Frozen Biopsies	4.84	[0.53, 9.15]	9
Gag	CD8+	Frozen Suspension	3.51	[-0.65, 7.67]	8
PMA/Iono	CD4+	Fresh	61.12	[49.14, 73.1]	13
PMA/Iono	CD4+	Frozen Biopsies	69.74	[62.78, 76.69]	13
PMA/Iono	CD4+	Frozen Suspension	58.30	[34.5, 82.1]	6
PMA/Iono	CD8+	Fresh	78.64	[70.01, 87.27]	15
PMA/Iono	CD8+	Frozen Biopsies	74.71	[66.13, 83.3]	15
PMA/Iono	CD8+	Frozen Suspension	68.41	[55.72, 81.1]	10
SEB	CD4+	Fresh	32.27	[26.9, 37.63]	13
SEB	CD4+	Frozen Biopsies	25.89	[23.05, 28.73]	13
SEB	CD4+	Frozen Suspension	26.69	[18.98, 34.4]	5
SEB	CD8+	Fresh	31.86	[26.73, 36.99]	17
SEB	CD8+	Frozen Biopsies	24.38	[20.42, 28.34]	16
SEB	CD8+	Frozen Suspension	27.52	[21.91, 33.13]	11

Table U: Cells expressing at least one cytokine (%), paired samples only

Condition	CellType	Tissue	Mean	95% CI	n
CEF	CD4+	Fresh	1.06	[-0.31, 2.43]	4
CEF	CD4+	Frozen Biopsies	1.15	[-0.78, 3.07]	4
CEF	CD4+	Frozen Suspension	1.48	[-0.16, 3.13]	4
CEF	CD8+	Fresh	0.89	[-0.13, 1.91]	5
CEF	CD8+	Frozen Biopsies	0.89	[-0.53, 2.32]	5
CEF	CD8+	Frozen Suspension	3.17	[-1.09, 7.43]	5
Gag	CD4+	Fresh	1.05	[-5.37, 7.46]	2
Gag	CD4+	Frozen Biopsies	2.16	[-19.25, 23.56]	2
Gag	CD4+	Frozen Suspension	0.89	[0.07, 1.72]	2
Gag	CD8+	Fresh	6.79	[-1.58, 15.16]	8
Gag	CD8+	Frozen Biopsies	4.80	[-0.21, 9.81]	8
Gag	CD8+	Frozen Suspension	3.51	[-0.65, 7.67]	8
PMA/Iono	CD4+	Fresh	57.69	[36.33, 79.05]	6
PMA/Iono	CD4+	Frozen Biopsies	65.97	[51.1, 80.85]	6
PMA/Iono	CD4+	Frozen Suspension	58.30	[34.5, 82.1]	6
PMA/Iono	CD8+	Fresh	73.80	[61.72, 85.89]	10
PMA/Iono	CD8+	Frozen Biopsies	69.01	[57.64, 80.38]	10
PMA/Iono	CD8+	Frozen Suspension	68.41	[55.72, 81.1]	10
SEB	CD4+	Fresh	33.72	[29.62, 37.83]	5
SEB	CD4+	Frozen Biopsies	26.64	[24.19, 29.08]	5
SEB	CD4+	Frozen Suspension	26.69	[18.98, 34.4]	5
SEB	CD8+	Fresh	31.57	[23.49, 39.65]	10
SEB	CD8+	Frozen Biopsies	23.46	[18.93, 27.98]	10
SEB	CD8+	Frozen Suspension	26.98	[20.83, 33.14]	10

Table V: T cell percentages

Condition	CellType	Mean	95% CI	n
Fresh	CD3 of total	2.82	[1.81, 3.82]	18
Frozen Biopsy	CD3 of total	1.80	[1.23, 2.38]	18
Frozen Suspension	CD3 of total	1.82	[1.37, 2.27]	17
Fresh	CD4 of CD3	39.14	[30.82, 47.45]	18
Frozen Biopsy	CD4 of CD3	37.76	[29.33, 46.18]	18
Frozen Suspension	CD4 of CD3	12.98	[5.12, 20.85]	17
Fresh	CD8 of CD3	40.87	[31.68, 50.07]	18
Frozen Biopsy	CD8 of CD3	40.31	[31.19, 49.43]	18
Frozen Suspension	CD8 of CD3	40.50	[30.63, 50.37]	17

Table W: T cell percentages, paired samples only

Condition	CellType	Mean	95% CI	n
Fresh	CD3 of total	2.72	[1.67, 3.76]	17
Frozen Biopsy	CD3 of total	1.81	[1.19, 2.42]	17
Frozen Suspension	CD3 of total	1.82	[1.37, 2.27]	17
Fresh	CD4 of CD3	38.24	[29.61, 46.88]	17
Frozen Biopsy	CD4 of CD3	37.40	[28.45, 46.35]	17
Frozen Suspension	CD4 of CD3	12.98	[5.12, 20.85]	17
Fresh	CD8 of CD3	41.67	[32.04, 51.3]	17
Frozen Biopsy	CD8 of CD3	40.75	[31.08, 50.42]	17
Frozen Suspension	CD8 of CD3	40.50	[30.63, 50.37]	17

Table X: CD3 percent, ANOVA with Tukey post-test

Comparisons	Estimate	Std. Error	Z value	p
Frozen Biopsy - Fresh	-1.01	0.48	-2.11	0.1
Frozen Suspension - Fresh	-1.00	0.49	-2.04	0.11
Frozen Suspension - Frozen Biopsy	0.02	0.49	0.04	1

Table Y: CD3 percent (paired samples only), repeated measures ANOVA with Tukey post-test

Comparisons	Estimate	Std. Error	Z value	p
Frozen Biopsy - Fresh	-0.91	0.32	-2.81	0.01
Frozen Suspension - Fresh	-0.90	0.32	-2.77	0.02
Frozen Suspension - Frozen Biopsy	0.01	0.32	0.04	1

Table Z: CD4 percent, ANOVA with Tukey post-test

Comparisons	Estimate	Std. Error	Z value	p
Frozen Biopsy - Fresh	-1.38	5.45	-0.25	0.97
Frozen Suspension - Fresh	-26.15	5.53	-4.73	4.56e-05
Frozen Suspension - Frozen Biopsy	-24.77	5.53	-4.48	1.27e-04

Table AA: CD4 percent (paired samples only), repeated measures ANOVA with Tukey post-test

Comparisons	Estimate	Std. Error	Z value	p
Frozen Biopsy - Fresh	-0.84	4.59	-0.18	0.98
Frozen Suspension - Fresh	-25.26	4.59	-5.50	1.03e-07
Frozen Suspension - Frozen Biopsy	-24.42	4.59	-5.31	2.54e-07

Table AB: CD8 percent, ANOVA with Tukey post-test

Comparisons	Estimate	Std. Error	Z value	p
Frozen Biopsy - Fresh	-0.56	6.22	-0.09	1
Frozen Suspension - Fresh	-0.37	6.31	-0.06	1
Frozen Suspension - Frozen Biopsy	0.19	6.31	0.03	1

Table AC: CD8 percent (paired samples only), repeated measures ANOVA with Tukey post-test

Comparisons	Estimate	Std. Error	Z value	p
Frozen Biopsy - Fresh	-0.92	1.32	-0.70	0.77
Frozen Suspension - Fresh	-1.17	1.32	-0.89	0.65
Frozen Suspension - Frozen Biopsy	-0.25	1.32	-0.19	0.98

Microbicide drug concentrations in cryopreserved tissue

Table AD: Drug concentration in cryopreserved tissues as a percentage of the concentration in fresh tissues

Drug	TissueType	InVitroConc	Condition	Mean	95% CI	n
dapivirine	Cervical	7.59 nM	Cryopreserved	95.84	[-34.2, 225.89]	3
dapivirine	Cervical	75.9 nM	Cryopreserved	59.64	[-6.8, 126.09]	3
dapivirine	Colorectal	0.759 nM	Cryopreserved	664.40	[-1331.69, 2660.48]	3
dapivirine	Colorectal	7.59 nM	Cryopreserved	43.88	[21.08, 66.68]	3
MK-2048	Cervical	10 µM	Cryopreserved	70.18	[8.23, 132.14]	3
MK-2048	Cervical	100 µM	Cryopreserved	97.21	[-18.52, 212.94]	3
MK-2048	Colorectal	1 µM	Cryopreserved	41.84	[8.05, 75.63]	3
MK-2048	Colorectal	10 µM	Cryopreserved	95.11	[40.41, 149.81]	3
tenofovir	Cervical	1.161 µM	Cryopreserved	2.58	[-2.69, 7.85]	3
tenofovir	Cervical	3.48 µM	Cryopreserved	36.75	[-66.66, 140.16]	3
