# Supplementary material for: Cryopreservation of human mucosal tissues
Source: PLoS One. 2018 Jul 30;13(7):e0200653. doi: 10.1371/journal.pone.0200653 (PMC6066204; doi:10.1371/journal.pone.0200653)
Supplement: S2 File — (ZIP) [file pone.0200653.s006.zip › data-overview-final.docx]

Overview of data

This document describes the data files.

data/clean/colorectal-vitrification-vs-cryopreservation-phenotyping.csv: Effect of cryopreservation or vitrification on phenotype of rectal biopsy cells.

| Variable | Values | Description |
| --- | --- | --- |
| TissueId | Four-digit ID numbers | Tissue donor ID number |
| Condition | Cryopreserved  Vitrified | Whether the biopsies were cryopreserved or vitrified |
| CellType | “n” = “negative”  “p” = “positive” | Type of cell |
| Percent | 0-100 | Percent of cells falling into this type |

data/clean/colorectal-vitrification-vs-cryopreservation-viacount.csv: Effect of cryopreservation or vitrification on rectal biopsy cell yield.

| Variable | Values | Description |
| --- | --- | --- |
| TissueId | Four-digit ID numbers | Tissue donor ID number |
| Condition | Cryopreserved Vitrified | Whether the biopsies were cryopreserved or vitrified |
| Viability | 0.51-0.87 | Viability (fraction, max 1) |
| ViableCount | 40000-8732000 | Number of cells |

data/clean/Dezzutti-Bushman-MK-2048.csv: Effect of cryopreservation on retention of microbicide drug level in explants.

| Variable | Values | Description |
| --- | --- | --- |
| TissueId | Donor ID codes | Tissue donor ID code |
| Explant | 1-16 | Explant number from this donor |
| Condition | Cryopreserved Fresh | Whether and how the biopsy was preserved |
| Concentration | 59.56-69164.53 | Measured concentration of drug recovered (units indicated in Units column) |
| LLOQ | 0.67-2.96 | Lower limit of quantitation |
| Drug | MK-2048 | Which drug was applied and measured |
| Units | pg/mg | Concentration of drug measured per mass of tissue |
| InVitroMicromolar | 1-100 | Concentration of drug applied to explant in vitro (micromolar) |
| TissueType | Cervical Colorectal | Anatomical type of tissue |

data/clean/Dezzutti-cervix-infection.csv: Effect of cryopreservation on HIV infection in cervical tissue.

| Variable | Values | Description |
| --- | --- | --- |
| Condition | 10% DMSO  10% DMSO + 50mM trehalose  6% DMSO + 5% EG + 50mM trehalose  7% DMSO 8% DMSO + 50mM trehalose  Fresh | Whether and how the biopsy was preserved |
| Replicate | 1-12 | Explant number from this donor |
| Day | 3-21 | Which day post infection the supernatant was collected |
| Hiv | 6.95-61814.16 | Concentration of HIV antigen detected in supernatant |
| TissueId | Donor ID codes | Tissue donor ID code |

data/clean/Dezzutti-rectum-infection.csv: Effect of cryopreservation on HIV infection in colorectal tissue.

| Variable | Values | Description |
| --- | --- | --- |
| Condition | 10% DMSO  10% DMSO + 50mM trehalose  6% DMSO + 5% EG + 50mM trehalose  7% DMSO 8% DMSO + 50mM trehalose  Fresh | Whether and how the biopsy was preserved |
| Replicate | 1-12 | Which explant from this donor |
| Day | 3-21 | Which day post infection the supernatant was collected |
| Hiv | 1-477890.41 | Concentration of HIV antigen detected in supernatant |
| TissueId | Donor ID codes | Tissue donor ID number |

data/clean/Dezzutti-Rohan-DPV-TFV.csv: Effect of cryopreservation on retention of microbicide drug level in explants.

| Variable | Values | Description |
| --- | --- | --- |
| Explant | 1-4 | Explant number from this donor |
| TissueId | CR 664A CR 673A CR 675A CR 675B CR 676 CVX 593 NDRI 196 NDRI 198A NDRI 199 NDRI 201B | Tissue donor ID number |
| Concentration | 0.002-37.63  “BMDL” = “below minimum detection limit” | Measured concentration of drug recovered (units indicated in Units column) |
| Condition | Cryopreserved  Fresh | Whether and how the biopsy was preserved |
| Units | ng/mg | Concentration of drug measured per mass of tissue |
| Drug | dapivirine  tenofovir | Which drug was applied and measured |
| TissueType | Cervical  Colorectal | Anatomical type of tissue |
| InVitroMicromolar | 0-3.48 | Concentration of drug applied to explant in vitro (micromolar) |

data/clean/Hughes-vagina-infection.csv: Effect of cryopreservation on HIV infection in vaginal tissue.

| Variable | Values | Description |
| --- | --- | --- |
| Condition | Cryopreservation  Formalin  Fresh  Vitrification | Whether and how the biopsy was preserved |
| Replicate | 1-3 | Explant number from this donor |
| Day | 1-20 | Which day post infection the supernatant was collected |
| Hiv | 204.01-11831200 | HIV as measured by luminescence from nanoluciferase |
| TissueId | 331-347 | Tissue donor ID number |

data/clean/Hughes-vagina-viability.csv: Effect of preservation on metabolic activity in vaginal tissue.

| Variable | Values | Description |
| --- | --- | --- |
| Condition | String describing how the explant was treated | Whether and how the biopsy was preserved |
| Wells | E.g. “A01” where letter corresponds to row and number to column | Well position ID |
| Fluorescence | 203.53-247117008 | Alamar blue-derived fluorescence |
| Rest | No rest  Rest | Whether or not the tissue was rested overnight |
| ReadingDay | Day 0  Day 1  Day 2 | Day on which alamar blue was measured |
| TissueId | 323-347 | Tissue donor ID number |
| Timepoint | 1.5-6 | Hours after addition of alamar blue reagent that fluorescence was measured |
| BiopsyDiameter | 2-5 | Diameter of biopsies (mm) |
| Cryopreservative | 10% DMSO  20%EG 20%DMSO  C1/C2/C3  ES/VS | Chemicals used for preservation |
| FreezingMethod | Mr. Frosty  Snap freeze | How the biopsy was frozen |
| VitrificationDevice | Aluminum foil, Copper foil,  Cryotissue, Cryovial dry,  Cryovial wet, Ova type M  Straw dry, Straw wet | Tool used to freeze the biopsy |
| PresoakLength | 0, 1.25, 10, 2.5, 20, 5, C1/C2/C3, ES/VS | How long the biopsy was soaked in preservation media (min) |
| HalfConcentrationVitrificationMedium | FALSE TRUE | Whether or not the biopsy was soaked in half concentration vitrification medium before transfer to full concentration |
| GradientThaw | FALSE TRUE | Whether or not the biopsy was thawed in a medium containing preservation chemicals |
| Standard | FALSE TRUE | Whether or not the biopsy was preserved in the way that came to be considered standard |

data/clean/Shacklett-Ferre-cytokines.csv: Effect of preservation on immune cell function in rectal tissue.

| Variable | Values | Description |
| --- | --- | --- |
| Ptid | 1247-7236 | Tissue donor ID number |
| HIV | Negative  Positive | Whether the donor has HIV infection |
| CellType | CD4+  CD8+ | Type of cell measured |
| Stimulation | CEF, DMSO, Gag, PMA/Iono, SEB | Reagent used to stimulate the cells |
| Tissue | Fresh GALT, Fresh PBMC, Frozen GALT Biopsies, Frozen GALT Suspension, Frozen PBMC | What type of sample and whether and how it was preserved |
| Cytokines | “7” = “CD107A”, “F” = “IFN-gamma”, “2” = “IL-2”, “M” = “MIP-1beta”, “T” = “TNF-alpha” | Which cytokines were produced |
| Percent | 0-100 | Percent of cells producing these cytokines |

data/clean/Shacklett-Ferre-proportions-T-cells.csv: Effect of preservation on T cell frequencies in rectal tissue.

| Variable | Values | Description |
| --- | --- | --- |
| Ptid | 1247-7236 | Tissue donor ID number |
| HIV | Negative  Positive | Whether the donor has HIV infection |
| Tissue | Fresh, Frozen Biopsy, Frozen Suspension | What type of sample and whether and how it was preserved |
| Stimulation | CEF, DMSO, Gag, PI, SEB | Reagent used to stimulate the cells |
| CellType | CD3 of total, CD4 of CD3, CD8 of CD3 | Type of cell measured |
| Percent | 0.29-83.16 | Percent of cells falling into this population |

data/clean/Shacklett-Ferre-yield-and-viability.csv: Effect of preservation on immune cell function in rectal tissue.

| Variable | Values | Description |
| --- | --- | --- |
| Ptid | 1247-7326 | Tissue donor ID number |
| Tissue | Fresh GALT, Fresh PBMC, Frozen GALT Biopsies, Frozen GALT Suspension, Frozen PBMC | What type of sample and whether and how it was preserved |
| HIV | Negative Positive | Whether the donor has HIV infection |
| SampleDate | Month/Day/Year | Date sample was processed |
| AssayDate | Month/Day/Year | Date assay was performed |
| CellNumber | 2.2-46 | Number of live cells by trypan blue (millions) |
| Viability | 53-100 | Viability (%, max 100) |
