# Supplementary material for: Cryopreservation of human mucosal tissues
Source: PLoS One. 2018 Jul 30;13(7):e0200653. doi: 10.1371/journal.pone.0200653 (PMC6066204; doi:10.1371/journal.pone.0200653)
Supplement: S2 File — (ZIP) [file pone.0200653.s006.zip › README.html]

README


# README

#### *Sean Hughes*

This directory holds the code and data used to generate the figures and statistics presented in the accompanying manuscript. This directory is assumed to be the working directory. A brief summary of the files follows:

- `data/` contains all of the data. The `raw/` sub-directory contains the raw files and the `clean/` subdirectory contains the files after processing. The various `.R` files in `data/` convert the raw data into processed forms.
- `functions/analysis-functions.R` has helper functions for common data aggregation and summary tasks.
- `functions/get-data.R` provides functions that load and sometimes pre-process data.
- `functions/infection-analysis-functions.R` contains functions for infection data.
- `functions/statistical-utils.R` has helper functions for consistent statistical tables
- `functions/style-functions.R` has helper functions for consistent styling of figures
- `figures/figure*.R` generates the images used in a particular figure. `figures/figures.R` sources all of the `figureX.R` files, updating all of the images.
- `statistics.Rmd` generates the statistical tables
- `source-all.R` sources all files necessary for the figures and statistics to be generated

To reproduce the analysis, first either open the `.Rproj` file or set the working directory to the folder this file is contained in. Then, to recreate all of the figures, `source("figures/figures.R")`. This will create all the figures as individual image files in `/figures/`. To recreate the statistical tables, knit the `statistics.Rmd` file with `knitr`. This will create `statistics.pdf` in the top level directory.

Figures, statistics, and this file were generated on 2018-05-30, using the following packages and versions:

```
## Session info -------------------------------------------------------------
```

```
##  setting  value                       
##  version  R version 3.4.1 (2017-06-30)
##  system   x86_64, mingw32             
##  ui       RTerm                       
##  language (EN)                        
##  collate  English_United States.1252  
##  tz       America/Los_Angeles         
##  date     2018-05-30
```

```
## Packages -----------------------------------------------------------------
```

```
##  package      * version   date       source        
##  acepack        1.4.1     2016-10-29 CRAN (R 3.4.4)
##  assertthat     0.2.0     2017-04-11 CRAN (R 3.4.3)
##  backports      1.1.2     2017-12-13 CRAN (R 3.4.3)
##  base         * 3.4.1     2017-06-30 local         
##  base64enc      0.1-3     2015-07-28 CRAN (R 3.4.1)
##  bindr          0.1.1     2018-03-13 CRAN (R 3.4.1)
##  bindrcpp     * 0.2       2017-06-17 CRAN (R 3.4.3)
##  broom        * 0.4.3     2017-11-20 CRAN (R 3.4.3)
##  cellranger     1.1.0     2016-07-27 CRAN (R 3.4.3)
##  checkmate      1.8.5     2017-10-24 CRAN (R 3.4.4)
##  cli            1.0.0     2017-11-05 CRAN (R 3.4.3)
##  cluster        2.0.6     2017-03-10 CRAN (R 3.4.1)
##  codetools      0.2-15    2016-10-05 CRAN (R 3.4.1)
##  colorspace     1.3-2     2016-12-14 CRAN (R 3.4.3)
##  compiler       3.4.1     2017-06-30 local         
##  crayon         1.3.4     2017-09-16 CRAN (R 3.4.3)
##  data.table     1.10.4-3  2017-10-27 CRAN (R 3.4.3)
##  datasets     * 3.4.1     2017-06-30 local         
##  devtools       1.13.5    2018-02-18 CRAN (R 3.4.3)
##  digest         0.6.15    2018-01-28 CRAN (R 3.4.3)
##  dplyr        * 0.7.4     2017-09-28 CRAN (R 3.4.3)
##  evaluate       0.10.1    2017-06-24 CRAN (R 3.4.3)
##  forcats      * 0.3.0     2018-02-19 CRAN (R 3.4.3)
##  foreign        0.8-69    2017-06-22 CRAN (R 3.4.1)
##  Formula      * 1.2-2     2017-07-10 CRAN (R 3.4.1)
##  ggplot2      * 2.2.1     2016-12-30 CRAN (R 3.4.3)
##  glue           1.2.0     2017-10-29 CRAN (R 3.4.3)
##  graphics     * 3.4.1     2017-06-30 local         
##  grDevices    * 3.4.1     2017-06-30 local         
##  grid           3.4.1     2017-06-30 local         
##  gridExtra      2.3       2017-09-09 CRAN (R 3.4.4)
##  gtable         0.2.0     2016-02-26 CRAN (R 3.4.3)
##  haven          1.1.1     2018-01-18 CRAN (R 3.4.3)
##  Hmisc        * 4.1-1     2018-01-03 CRAN (R 3.4.4)
##  hms            0.4.2     2018-03-10 CRAN (R 3.4.3)
##  htmlTable      1.11.2    2018-01-20 CRAN (R 3.4.4)
##  htmltools      0.3.6     2017-04-28 CRAN (R 3.4.3)
##  htmlwidgets    1.0       2018-01-20 CRAN (R 3.4.4)
##  httr           1.3.1     2017-08-20 CRAN (R 3.4.3)
##  jsonlite       1.5       2017-06-01 CRAN (R 3.4.3)
##  knitr          1.20      2018-02-20 CRAN (R 3.4.3)
##  lattice      * 0.20-35   2017-03-25 CRAN (R 3.4.1)
##  latticeExtra   0.6-28    2016-02-09 CRAN (R 3.4.4)
##  lazyeval     * 0.2.1     2017-10-29 CRAN (R 3.4.3)
##  lubridate      1.7.2     2018-02-06 CRAN (R 3.4.3)
##  magrittr       1.5       2014-11-22 CRAN (R 3.4.3)
##  MASS         * 7.3-49    2018-02-23 CRAN (R 3.4.3)
##  Matrix         1.2-12    2017-11-16 CRAN (R 3.4.3)
##  memoise        1.1.0     2017-04-21 CRAN (R 3.4.3)
##  methods      * 3.4.1     2017-06-30 local         
##  mnormt         1.5-5     2016-10-15 CRAN (R 3.4.1)
##  modelr         0.1.1     2017-07-24 CRAN (R 3.4.3)
##  multcomp     * 1.4-8     2017-11-08 CRAN (R 3.4.4)
##  munsell        0.4.3     2016-02-13 CRAN (R 3.4.3)
##  mvtnorm      * 1.0-7     2018-01-26 CRAN (R 3.4.3)
##  nlme         * 3.1-131.1 2018-02-16 CRAN (R 3.4.3)
##  nnet           7.3-12    2016-02-02 CRAN (R 3.4.1)
##  pander       * 0.6.1     2017-08-06 CRAN (R 3.4.3)
##  parallel       3.4.1     2017-06-30 local         
##  pillar         1.2.1     2018-02-27 CRAN (R 3.4.3)
##  pkgconfig      2.0.1     2017-03-21 CRAN (R 3.4.3)
##  plater       * 1.0.1     2017-06-26 CRAN (R 3.4.4)
##  plyr           1.8.4     2016-06-08 CRAN (R 3.4.3)
##  psych          1.7.8     2017-09-09 CRAN (R 3.4.3)
##  purrr        * 0.2.4     2017-10-18 CRAN (R 3.4.3)
##  R6             2.2.2     2017-06-17 CRAN (R 3.4.3)
##  RColorBrewer   1.1-2     2014-12-07 CRAN (R 3.4.1)
##  Rcpp           0.12.15   2018-01-20 CRAN (R 3.4.3)
##  readr        * 1.1.1     2017-05-16 CRAN (R 3.4.3)
##  readxl       * 1.0.0     2017-04-18 CRAN (R 3.4.3)
##  reshape2       1.4.3     2017-12-11 CRAN (R 3.4.3)
##  rlang          0.2.0     2018-02-20 CRAN (R 3.4.3)
##  rmarkdown      1.9       2018-03-01 CRAN (R 3.4.3)
##  rpart          4.1-13    2018-02-23 CRAN (R 3.4.3)
##  rprojroot      1.3-2     2018-01-03 CRAN (R 3.4.3)
##  rstudioapi     0.7       2017-09-07 CRAN (R 3.4.3)
##  rvest          0.3.2     2016-06-17 CRAN (R 3.4.3)
##  sandwich       2.4-0     2017-07-26 CRAN (R 3.4.4)
##  scales         0.5.0     2017-08-24 CRAN (R 3.4.3)
##  splines        3.4.1     2017-06-30 local         
##  stats        * 3.4.1     2017-06-30 local         
##  stringi        1.1.6     2017-11-17 CRAN (R 3.4.2)
##  stringr      * 1.3.0     2018-02-19 CRAN (R 3.4.3)
##  survival     * 2.41-3    2017-04-04 CRAN (R 3.4.1)
##  TH.data      * 1.0-8     2017-01-23 CRAN (R 3.4.4)
##  tibble       * 1.4.2     2018-01-22 CRAN (R 3.4.3)
##  tidyr        * 0.8.0     2018-01-29 CRAN (R 3.4.3)
##  tidyverse    * 1.2.1     2017-11-14 CRAN (R 3.4.3)
##  tools          3.4.1     2017-06-30 local         
##  utils        * 3.4.1     2017-06-30 local         
##  withr          2.1.1     2017-12-19 CRAN (R 3.4.3)
##  xml2           1.2.0     2018-01-24 CRAN (R 3.4.3)
##  yaml           2.1.18    2018-03-08 CRAN (R 3.4.3)
##  zoo            1.8-1     2018-01-08 CRAN (R 3.4.4)
```
